# Effects of probiotic type, dose and treatment duration on irritable bowel syndrome diagnosed by Rome III criteria: a meta-analysis

**DOI:** 10.1186/s12876-016-0470-z

**Published:** 2016-06-13

**Authors:** Yan Zhang, Lixiang Li, Chuanguo Guo, Dan Mu, Bingcheng Feng, Xiuli Zuo, Yanqing Li

**Affiliations:** Department of Gastroenterology, Laboratory of Translational Gastroenterology, Qilu Hospital, Shandong University, 107 Wenhuaxi Road, Jinan, 250012 Shandong Province China

**Keywords:** Probiotics, Irritable bowel syndrome, Meta-analysis

## Abstract

**Background:**

Irritable bowel syndrome (IBS) is one of the most common functional gastroenterological diseases, affecting 11.2 % of people worldwide. Previous studies have shown that probiotic treatment may benefit IBS patients. However, the effect of probiotics and the appropriate type, dose, and treatment duration for IBS are still unclear. The aim of the current study was to assess the efficacy of different probiotic types, doses and treatment durations in IBS patients diagnosed by Rome III criteria via a meta-analysis of randomized controlled trials (RCTs).

**Methods:**

Medline, EMBASE, and the Cochrane Central Register of Controlled Trials up to October 2015 were searched. RCTs including comparisons between the effects of probiotics and placebo on IBS patients diagnosed by Rome III criteria were eligible. Dichotomous data were pooled to obtain the relative risk (RR) with a 95 % confidence interval (CI), whereas continuous data were pooled using a standardized mean difference (SMD) with a 95 % CI.

**Results:**

Twenty-one RCTs were included in this meta-analysis. Probiotic therapy was associated with more improvement than placebo administration in overall symptom response (RR: 1.82, 95 % CI 1.27 to 2.60) and quality of life (QoL) (SMD: 0.29, 95 % CI 0.08 to 0.50), but not in individual IBS symptoms. Single probiotics, a low dose, and a short treatment duration were more effective with respect to overall symptom response and QoL. No differences were detected in individual IBS symptoms in the subgroup analyses.

**Conclusion:**

Probiotics are an effective pharmacological therapy in IBS patients. Single probiotics at a low dose and with a short treatment duration appear to be more effective in improving overall symptom response and QoL, but more evidence for these effects is still needed.

**Electronic supplementary material:**

The online version of this article (doi:10.1186/s12876-016-0470-z) contains supplementary material, which is available to authorized users.

## Background

Irritable bowel syndrome (IBS) is characterized by abdominal pain and alterations in bowel habits. It affects 11.2 % of people worldwide [1] and is regarded as one of the most common functional gastroenterological diseases [2]. Although the exact pathophysiology underlying IBS is still not fully understood, chronic low-grade mucosal inflammation, alterations in gut epithelial and immune function, and visceral hypersensitivity caused by alterations in intestinal microbiota have been shown to be associated with IBS [3–5]. The current therapeutic options for IBS treatment include low-dose antidepressants, spasmolytics, and 5-HT3 antagonists. However, IBS patients often have variant response to these therapies, and they are also associated with several complications [6–9]. Antidepressant treatment often causes severe problems, including weight gain, and cannot be tolerated by many patients. Spasmolytics and 5-HT3 antagonists are ineffective for some people, and they may even worsen the symptoms of IBS [7]. Moreover, long-term use of these medications in IBS patients can increase the occurrence of various adverse effects [10].

New therapeutic options with the potential to alter intestinal microbiota have recently been identified and include the low fermentable, oligo-, di-, monosaccharides, and polyols (FODMAP) diet [11], antibiotics [12], and probiotics. Probiotics, defined as “live microorganisms that, when administered in adequate amounts, confer a health benefit on the host” [13], have the potential to influence the intestinal microbiota. Probiotics may affect intestinal barrier function and exert anti-inflammatory actions [10]. To date, many clinical studies have investigated the effects of probiotics in IBS patients, and more than half of these studies demonstrated that probiotic administration is effective in IBS patients [14]. Due to differences in the study designs (size of the study, duration of the treatment), probiotic doses, and strains used, clinical studies addressing the efficacy of probiotics in IBS are difficult to compare [15]. Several systematic reviews and meta-analyses on the effects of probiotics in IBS patients have been generated, and the majority of results demonstrated that the use of probiotics was beneficial in IBS patients [14, 16–20]. Despite these findings, some issues concerning probiotic treatment in IBS patients persist; specifically, the type of probiotic used in different studies varied, combination probiotics and single probiotics were both used, and the doses and treatment durations were also different between studies. Rome III criteria [21], based on Rome II criteria, applied 10 years ago, have been used more extensively than Rome I/II and Manning criteria [22–24]. The Rome II subtyping using multiple criteria was complex and difficult to use in practice. Compared with Rome II criteria, Rome III criteria require a lower frequency of IBS symptoms and focus more on recent symptom severity. The latter change may lead to increased compliance in and comparability between patients enrolled in clinical trials [2]. Previous studies also indicated that the Rome III assessment may more accurately reflect the burden of disease and epidemiological features than the Rome II criteria [25]. However, no meta-analysis based on studies using the Rome III criteria has been performed to date. Further investigations are clearly needed to establish optimal treatment regimens (the most effective probiotic species and strains, individual or mixture administration), as well as to identify subgroups of patients most likely to benefit from these treatments [26].

In this study, we conducted a meta-analysis to assess the efficacy of different types of probiotics in IBS patients with the Rome III criteria serving as the diagnostic criteria. We also analysed the effects of different doses and treatment durations in IBS patients.

## Methods

### Literature search

We systematically searched the Medline, EMBASE, and Cochrane Central Register of Controlled Trials databases up to October 2015 for studies that investigated the efficacy of probiotic therapy in IBS patients. We used the terms “probiotics” and “irritable bowel syndrome” both as medical subject heading (Mesh) and free text terms. The exact search strategy in Medline was ("probiotics"[MeSH Terms] OR "probiotics"[All Fields]) AND ("irritable bowel syndrome"[MeSH Terms] OR "irritable bowel syndrome"[All Fields]). All eligible studies were retrieved, and the bibliographies were manually checked to identify additional potential studies.

### Inclusion and exclusion criteria

Studies were considered eligible if they met the following criteria: (1) the studies were randomized controlled trials (RCTs) that compared probiotics with placebo; (2) the diagnosis of IBS was made according to the Rome III criteria; (3) the treatment duration was >7 days; and (4) dichotomous data on the overall syndrome response to the therapy or continuous score data on the effect on individual IBS symptoms or quality of life (QoL) could be extracted or obtained from the authors. The exclusion criteria were as follows: (1) studies with only an abstract; (2) studies in which probiotics were mixed with other drugs; (3) studies in which data were still unavailable after contacting the authors; and (4) studies in which the control group received probiotics.

### Data extraction

Two authors independently extracted the data from each of the eligible articles according to the inclusion and exclusion criteria. The data included the first author, publication year, country, criteria used to diagnose IBS, dose of probiotic, treatment duration and follow-up time, number of patients, mean after-treatment scores along with the standard deviation (s.d.) of individual IBS symptoms (abdominal pain and bloating), and QoL. All data were extracted for intention to treat (ITT) analysis, whereby all dropouts were assumed to be treatment failures. Only the data associated with the longest duration of therapy and largest dose were used to compare the efficacy between probiotic types.

### Assessment of risk of bias

Two authors independently performed the assessment of bias risk, with disagreements resolved by discussion. The risk of bias was assessed as described in the Cochrane handbook [27] by recording the method used to generate the randomization schedule and the method used for allocation concealment, whether blinding was implemented, the completeness of follow-up, whether there was evidence of selective reporting of outcomes, and other biases.

### Statistical analysis

The pooled relative risk (RR) and corresponding 95 % confidence interval (CI) were calculated in the meta-analysis to evaluate the effect of the overall symptom response of IBS patients after treatment. The standardized mean difference (SMD) and 95 % CI were used to evaluate individual IBS symptoms and QoL. The I^2^ statistic was calculated to quantify the proportion of the total variation due to heterogeneity, and an I^2^ value of > 50 % indicated significant heterogeneity among studies. A random-effects model [28] was utilized to provide a more conservative estimate of the effects of probiotic treatment, assuming heterogeneity of treatment effects across studies. Subgroup analyses according to probiotic type, dose, and treatment duration were performed for the assessment of the effects on the overall response, individual IBS symptoms, and QoL. Sensitivity analyses were performed by omitting one study at a time and analysing the remaining studies to assess whether the results were excessively influenced by any single study. The possibility of publication bias was assessed by visual inspection of a funnel plot, and the Egger test was also performed to assess the possibility of publication bias [29]. A *P* value of < 0.05 was considered statistically significant. All statistical analyses were conducted using Stata Statistical Software: Release 12 (StataCorp LP; College Station, TX).

## Results

### Studies included in the meta-analysis

**Fig. 1:**
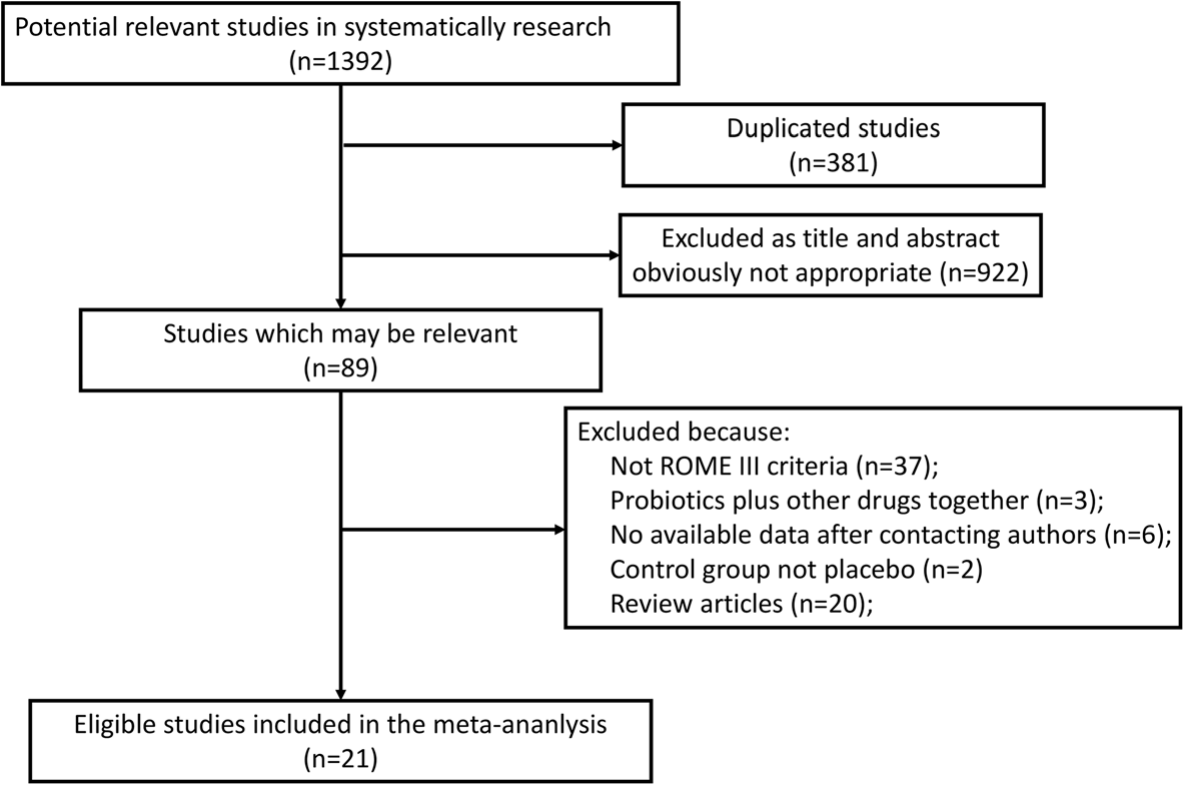
Flow diagram of assessment of studies identified in the meta-analysis

A total of 1392 publications were initially retrieved using our search strategy. Of these publications, 21 were included in the current meta-analysis [30–50]. The flow chart of search history is presented in Fig. 1. Agreements between the reviewers regarding the assessment of trial eligibility were ideal (kappa statistic = 0.88). The details of the RCTs included in the analysis are presented in Table 1.

**Table 1:** Characteristics of the randomized controlled trials of probiotics vs. placebo in irritable bowel syndrome

Study	Country	Recruitment	Sample size/probiotic	Probiotic	Dosage (CFU/D)	Duration of treatment (W)	Outcome measure
Sinn *et al.* [30]	Korea	Single-centre	40/20	*L. acidophilus-SDC 2012, 2013*	4 × 10^9^	4	Response (reduction scores)
Hong *et al.* [31]	Korea	Single-centre	70/36	*Bifidobacterium bifidum BGN4; Bifidobacterium lactis AD011; Lactobacillus acidophilus AD031; and Lactobacillus casei IBS041*	4 × 10^10^	8	Response (reduction scores)
Guglielmetti *et al.* [32]	Germany	Multi-centre	122/60	*Bifidobacterium bifidum* MIMBb75	10^9^	4	Response (>50 % relief); Pain/bloating (a 7-point Likert scale)
							QoL (SF-12)
Michail *et al.* [33]	USA	Single-centre	24/15	*VSL #3*	9 × 10^10^	8	Pain/bloating (GSRS)
							QoL (QoL questionnaires)
Ringel *et al.* [34]	USA	Single-centre	33/17	*L-NCFM and B-LBi07*	2 × 10^11^	8	Response (relief of symptoms)
Dapoigny *et al.* [35]	France	Multi-centre	50/25	*L. casei variety rhamnosus (*LCR35*)*	6 × 10^8^	4	Response (reduction scores)
							Pain/bloating (VAS)
							QoL (GIQLI)
Ducrotté *et al.* [36]	India	Multi-centre	214/108	*L. plantarum 299v (*DSM9843*)*	10^10^	4	Response (good and excellent overall efficacy)
							Pain/bloating (VAS)
Cui *et al.* [37]	China	Single-centre	60/37	*Bifidobacterium longum and Lactobacillus acidophilus*	6 × 10^7^	4	Pain/bloating (rating score)
Cha *et al.* [38]	Korea	Single-centre	50/25	*Lactobacillus acidophilus, Lactobacillus plantarum, Lactobacillus rhamnosus, Bifidobacterium breve, Bifidobacterium lactis, Bifidobacterium longum, and Streptococcus thermophiles*	10^10^	8	Response (relief of symptoms)
							Pain/bloating (VAS)
							QoL (a 5-point Likert scale)
Amirimani *et al.* [39]	Iran	Multi-centre	72/41	*Lactobacillus reuteri (*Biogaia®*)*	10^11^	4	Pain/bloating (questionnaire)
Begtrup *et al.* [40]	Denmark	Single-centre	131/67	*Lactobacillus para- casei ssp paracasei F19, Lactobacillus acidophilusLa5 and Bifidobacterium Bb12*	5 × 10^10^	24	Response (relief of symptoms)
							Pain/bloating (GSRS)
							QoL (HRQOL-questionnaire)
Ko *et al.* [41]	Korea	Single-centre	26/14	*Probiotics mixture containing 7 species (Lactobacillus acidophilus, Lactobacillus plantarum, Lactobacillus rhamnosus, Bifidobacterium breve, Bifidobacterium lactis, Bifidobacterium longum, and Streptococcus thermophiles)*	10^10^	8	Response (reduction scores)
							Pain/bloating (VAS)
							QoL (a 5-point Likert scale)
Roberts *et al.* [42]	UK	Single-centre	179/88	*Bifidobacterium lactis, S. thermophilus and L. bulgaricus*	2.5 × 10^10^	12	Response (relief of symptoms)
							Pain/bloating (a 6-point Likert scale)
							QoL (the Birmingham Symptom Score)
Yoon *et al.* [43]	Korea	Single-centre	49/25	*Bifidobacterium bifidum, Bifidobacterium lactis, Bifidobacterium longum, Lactobacillus acidophilus, Lactobacillus rhamnosus, and Streptococcus thermophiles.*	10^10^	4	Response (relief of symptoms)
							Pain/bloating (a 10-point numerical scale)
Abbas *et al.* [44]	Pakistan	Single-centre	72/37	*S. boulardii*	3 × 10^9^	6	Pain/Bloating (a 4-point numerical scale)
Lorenzo *et al.* [45]	Spain	Multi-centre	71/47	*L. plantarum and P. acidilactici (I.31)*	3 × 10^9^--6 × 10^9^	6	Response (relief of symptoms)
							QoL (a standardized score ranging from 1–100)
Ludidi *et al.* [46]	Netherlands	Single-centre	40/21	*Bifidobacterium lactis, Lactobacillus casei, Lactobacillus salivarius, Lactococcus lactis, Lactobacillus acidophilus, and Lactobacillus rhamnosus*	5 × 10^9^	6	Response (MMS)
							Pain/bloating (a 5-point Likert scale)
Urgesi *et al.* [47]	Italy	Single-centre	52/26	*Bacillus coagulans (Colinox®)*	4.5 × 10^9^	4	Response (a 4-point Likert scale)
							Pain/bloating (VAS)
Rogha *et al.* [48]	Iran	Single-centre	56/23	*Bacillus coagulans*	4.5 × 10^8^	12	Pain/bloating (a 7-point numeric scale)
Sisson *et al.* [49]	UK	Single-centre	186/124	*Symprove containing four strains of bacteria: Lactobacillus rhamnosus, Lactobacillus plantarum, Lactobacillus acidophilus, and Enterococcus faecium*	10^10^	12	Response (IBS-SSS)
							Pain/bloating (IBS-SSS)
							QoL (a 100-point numerical scale)
Wong *et al.* [50]	Singapore	Single-centre	42/20	*VSL #3*	4.5 × 10^11^	6	Pain/bloating (SBDQ)

*GSRS* gastrointestinal symptom rating scale, *VAS* visual analogue scale, GIQLI gastrointestinal quality of life index, HRQOL questionnaire health-related quality of life questionnaire, MMS mean symptom composite score, IBS-SSS irritable bowel syndrome-symptom severity score, SBDQ standardized bowel disease questionnaire

Additional files 1 and 2 in the supplementary material demonstrates the risk of bias for all studies assessed using the Cochrane Collaboration tool. Three studies did not describe the details of the sequence generation process [35, 37, 46], and 7 studies did not describe the method of concealment [30, 33, 34, 37–39, 48], leading to an unclear risk of selection bias. The risk of blinding of participants and personnel and risk of outcome assessment were low. One study did not use ITT analysis [48], leading to an elevated risk of attrition bias. All studies exhibited a low risk of reporting bias. Wong et al. reported that the higher anxiety reported by patients taking probiotics may influence the study results, leading to an unclear risk of other bias [50].

### Effects on overall symptom response in IBS patients

Sixteen RCTs [30–32, 34–38, 40–43, 45–47, 49], including 17 comparisons of the overall symptom response to probiotics versus placebo in the treatment of IBS patients, were identified. One of these RCTs, namely, the study by Lorenzo et al., examined two different dose groups [45].

The overall symptom response was the primary efficacy end-point in most studies. Overall symptom response was defined as a > 50 % reduction in IBS pain and discomfort or adequate relief of IBS symptoms for > 50 % of the time in 7 of 15 studies. Other definitions included an improvement of ≥ 50 points in the global IBS-symptom severity score (IBS-SSS), global relief of IBS symptoms, or good and excellent overall efficacy.

**Fig. 2:**
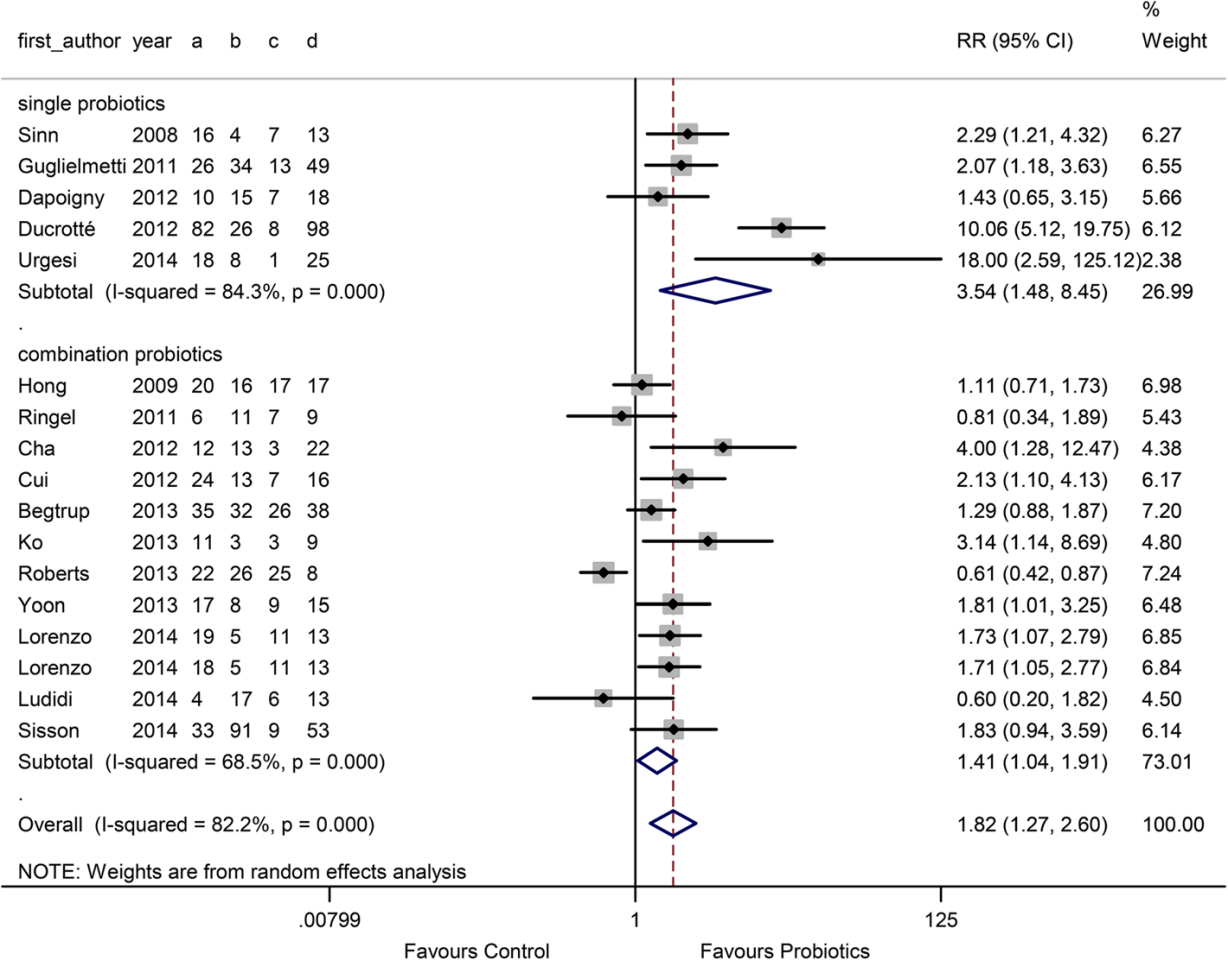
Forest plot of effect on overall symptom response of IBS patients to probiotics: subgroup of probiotics typeᅟ

A total of 700 IBS patients were allocated to the probiotics group, whereas 575 IBS patients constituted the control group. The overall symptom response rate was 53.3 % in the probiotics group and 27.7 % in the control group. The RR of overall symptom response was significantly higher in the probiotics group (1.82, 95 % CI 1.27 to 2.60), and this group also had a significant degree of heterogeneity (I^2^ = 82.2 %, *P* < 0.001) (Fig. 2).

**Fig. 3:**
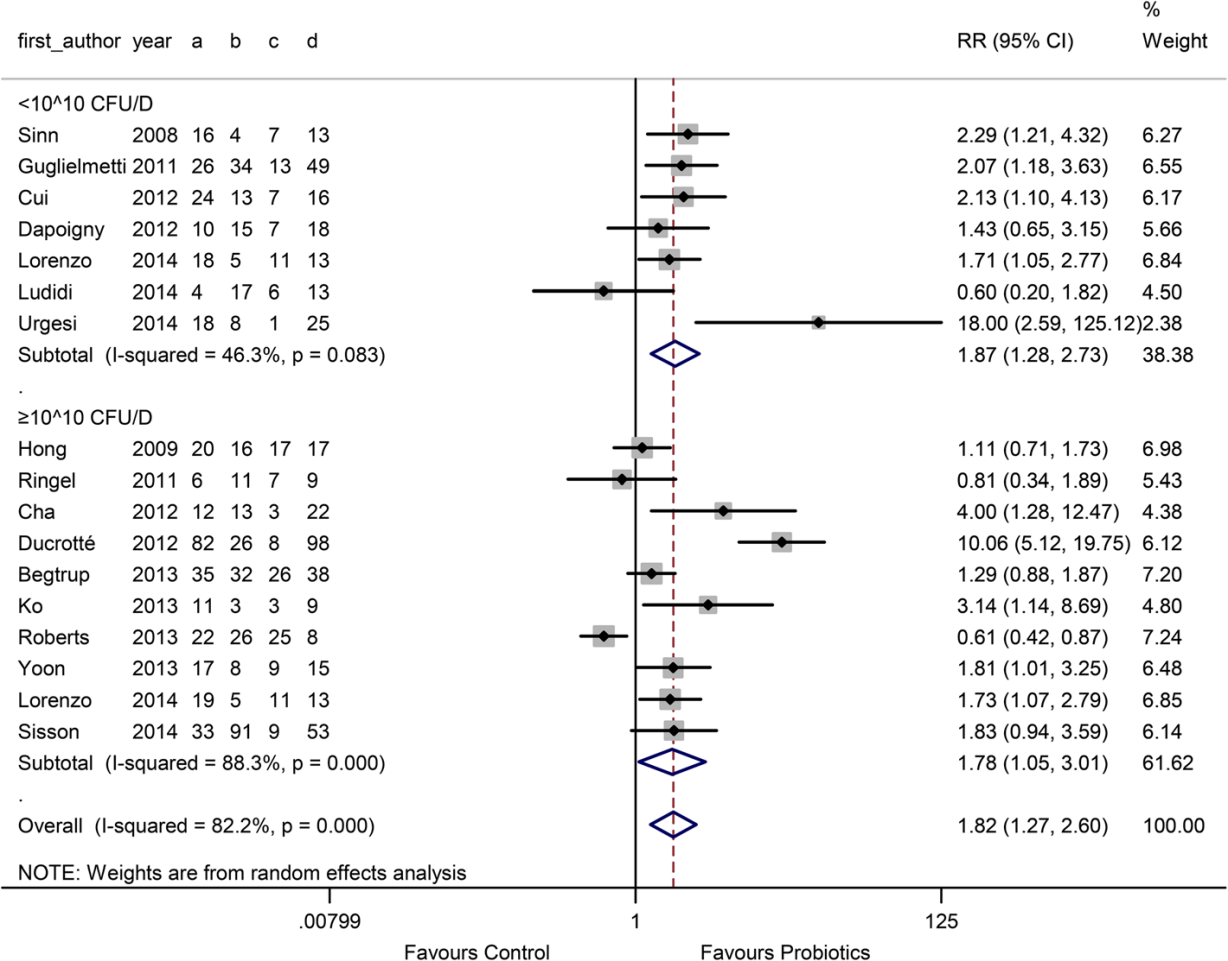
Forest plot of effect on overall symptom response of IBS patients to probiotics: subgroup of probiotics doseᅟ

**Fig. 4:**
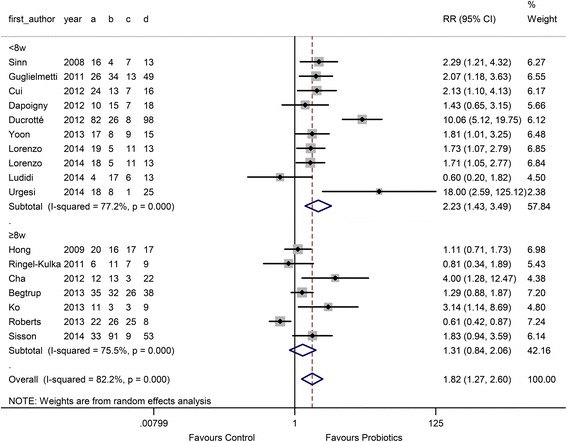
Forest plot of effect on overall symptom response of IBS patients to probiotics: subgroup of probiotics duration

In the probiotic type subgroup, 5 single probiotic and 12 combination probiotic comparisons were performed. The RR in the single and combination probiotics subgroups was 3.54 (95 % CI 1.48 to 8.45) and 1.41 (95 % CI 1.04 to 1.91), respectively, as shown in Fig. 2. There was high heterogeneity between studies (I^2^ = 84.3 %, *P* < 0.001 and 68.5 %, *P* < 0.001). In the probiotic dose subgroup, 7 comparisons used a low dose (< 10^10^ CFU/D) and 10 comparisons used a high dose (≥ 10^10^ CFU/D). The RR of the low-dose group was 1.87 (95 % CI 1.28 to 2.73), and the RR of the high-dose group was 1.78 (95 % CI 1.05 to 3.01) (Fig. 3). In the duration subgroup, 10 comparisons used a short treatment duration (<8 w), whereas 7 comparisons evaluated a relatively long duration (≥8 w). The RR of the short duration was 2.23 (95 % CI 1.43 to 3.49), and the RR of the long duration was 1.31 (95 % CI 1.27 to 2.60) (Fig. 4).

Funnel plot asymmetry suggested the existence of potential publication bias (Egger test, *P* = 0.04) (Additional file 3).

### Effects on abdominal pain in IBS patients

A total of 13 studies with 13 comparisons were included in the comparison of the effects of probiotics on abdominal pain [32, 33, 38–41, 43, 44, 46–50]. A total of 485 IBS patients were included in the probiotics group, and 404 IBS patients constituted the control group. Of the 13 included studies, 2 studies used a 100-mm visual analogue scale (VAS) measurement, 3 studies used a 7-point Likert scale, 2 studies used a 5-point Likert scale, and the remainder of the studies used another measurement or declared a lack of measurement. High heterogeneity existed between studies (I^2^ = 85.7 %, *P* < 0.001).

In the comparison of probiotics versus placebo, the overall SMD was −0.25 (95 % CI −0.62 to 0.13) (Additional file 4). Probiotic use was not associated with an improvement in abdominal pain compared with placebo. No significant differences were found for different probiotic types, doses, or treatment durations. There was no significant funnel plot asymmetry observed (Egger test, *P* = 0.90), suggesting no evidence of publication bias or other small-study effects (Additional file 5).

### Effects on bloating in IBS patients

Bloating is another severe symptom in IBS patients, and studies have suggested that gas production may be associated with probiotic use in IBS patients [51]. Thirteen studies with 13 comparisons were included in the comparison of the effect of probiotics on bloating, with 492 individuals allocated to the probiotics group and 398 individuals allocated to the control group [32–34, 38–41, 43, 44, 46, 47, 49, 50]. The measurements used to assess bloating were the same as those used to evaluate abdominal pain. Nine studies used combination probiotics, and 5 studies used single probiotics. The overall SMD was −0.19 (95 % CI −0.45 to 0.08), with high heterogeneity (I^2^ = 72.2 %, *P* < 0.001) (Additional file 6). No statistically significant differences were detected with respect to probiotic type, dose, or treatment duration. No significant funnel plot asymmetry (Egger test, *P* = 0.963) was observed, suggesting no evidence of publication bias or other small study effects (Additional file 7).

### Effects on QoL

IBS greatly impacts the QoL of IBS patients, and the degree of alteration in the quality of life is closely related to the severity of IBS in individual patients [52]. A recent study revealed that the QoL impact of severe IBS was similar to that of Class 3 congestive heart failure and rheumatoid arthritis [53]. Nine studies were included in the comparison of effects on QoL, with 364 subjects in the probiotics group and 265 subjects in the control group. Numeric score assessments included the SF-12, a 5-point Likert scale, and SMD was used to assess the effects of probiotics on QoL.

**Fig. 5:**
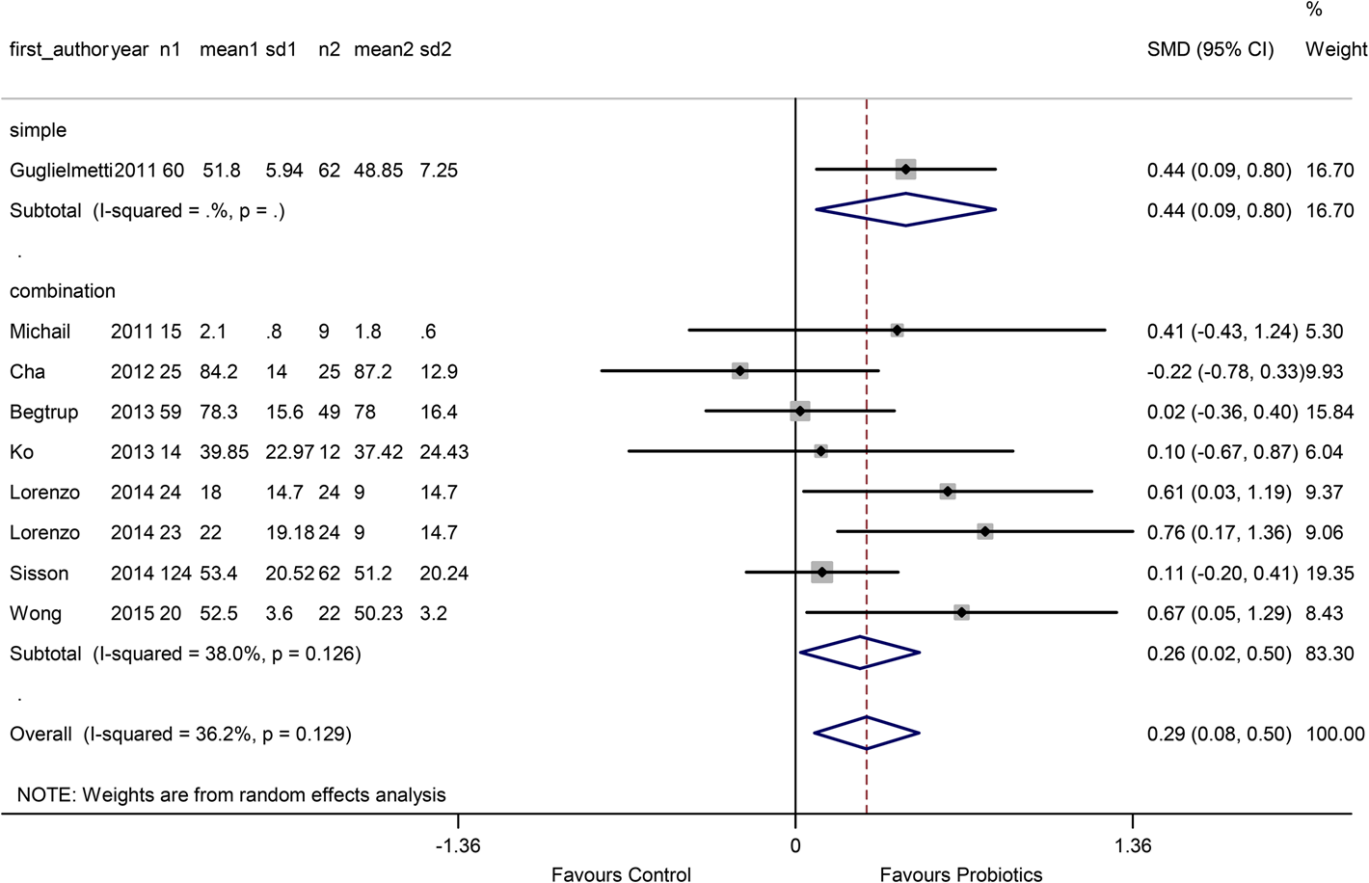
Forest plot of effect on QoL of IBS patients to probiotics: subgroup of probiotics type

The overall SMD was 0.29 (95 % CI 0.08 to 0.50), and the heterogeneity was low (I^2^ = 36.2 %). In the probiotic type subgroup, only 1 study used a single probiotic and the remaining 8 studies used combination probiotics. The RR of the combination probiotics was 0.26 (95 % CI 0.02 to 0.50), and the RR of the single probiotics was 0.44 (95 % CI 0.09 to 0.80), as shown in Fig. 5.

**Fig. 6:**
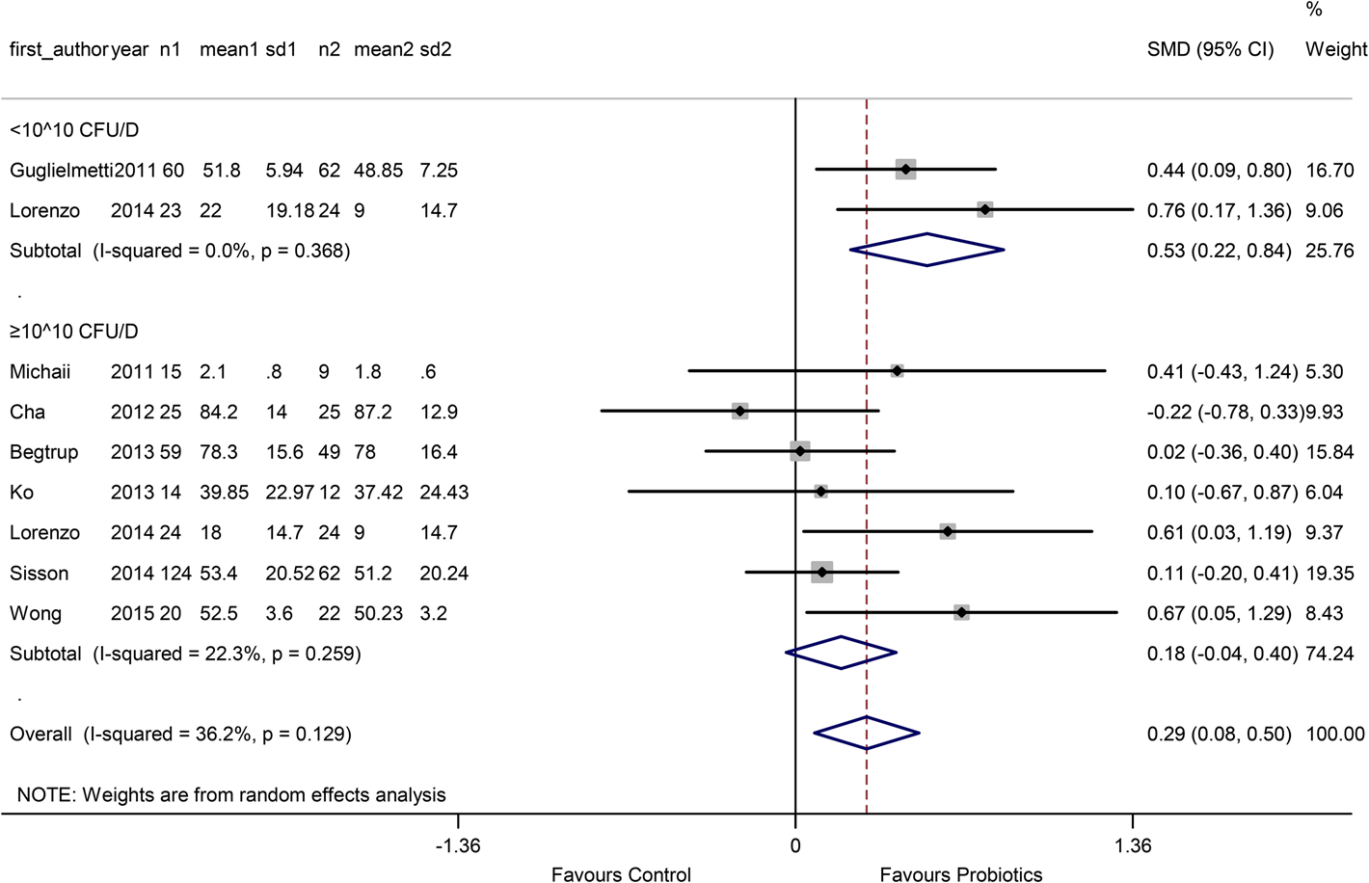
Forest plot of effect on QoL of IBS patients to probiotics: subgroup of probiotics dose

In the dose subgroup, 2 comparisons used a low dose (< 10^10^ CFU/D) and 7 comparisons used a high dose (≥ 10^10^ CFU/D). The SMD of the low dose was 0.53 (95 % CI 0.22 to 0.84), and the SMD of the high dose was 0.18 (95 % CI −0.04 to 0.40) (Fig. 6).

**Fig. 7:**
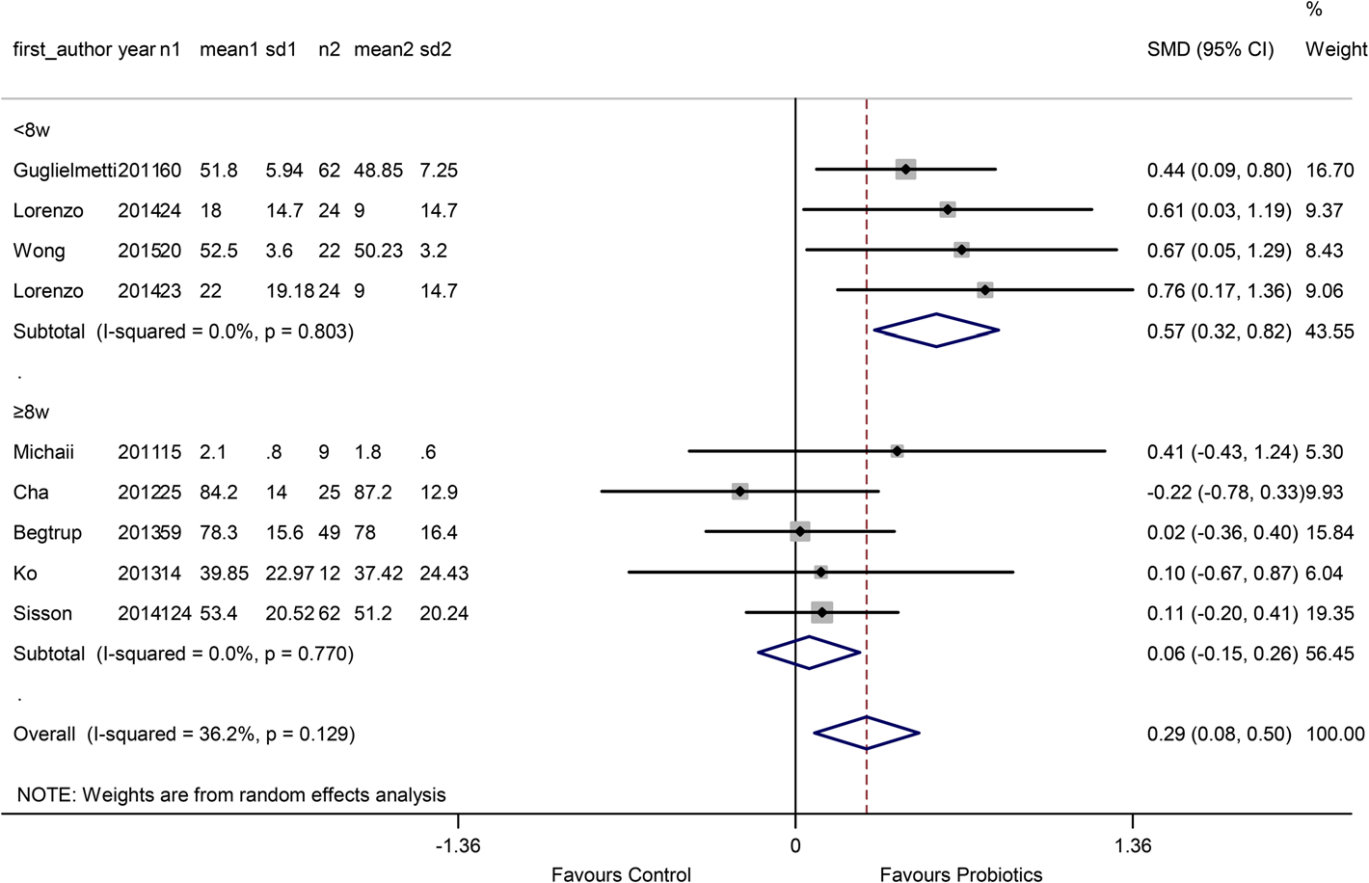
Forest plot of effect on QoL of IBS patients to probiotics: subgroup of probiotics duration

In the treatment duration subgroup, 4 comparisons used a short treatment duration (<8 w) and 5 comparisons used a relatively long duration (≥8 w). The RR of the short duration was 0.57 (95 % CI 0.32 to 0.82), and the RR of the long duration was 0.06 (95 % CI −0.15 to 0.26) (Fig. 7).

There was no significant funnel plot asymmetry detected (Egger test, *P* = 0.38), suggesting no evidence of publication bias or other small study effects (Additional file 8).

## Discussion

This meta-analysis indicated that probiotic use could significantly improve the overall symptom response and QoL in IBS patients compared with placebo. These results were consistent with those of previous systematic reviews that included other diagnostic criteria [14, 19, 20], which suggested that probiotics were effective in treating IBS. No significant differences were found in the relief of individual IBS symptoms (abdominal pain and bloating) between probiotics and placebo. This result is inconsistent with previous studies [14]. Previous studies suggested that combination probiotics could affect abdominal pain and bloating, but the individual probiotics *Lactobacilli* and *Bifidobacteria* were not effective [14, 19]. The larger proportion of *Lactobacilli* and *Bifidobacteria* in probiotics used in the studies included in this meta-analysis may explain the contradictory results on abdominal pain and bloating. The Rome III criteria constitute a useful tool with which to diagnose IBS, but the quantification of individual IBS symptoms is still subjective. The different diagnostic criteria and methods of quantifying individual IBS symptoms may contribute to the inconsistent reports on the efficacy of probiotic supplementation in treating individual IBS symptoms.

In the current meta-analysis, there were more studies that used combination probiotics compared with single probiotics. Only one study using single probiotics was included in the QoL assessment. Our results suggested that single probiotics appeared to be more effective in the overall symptom response (*P* = 0.04), but not QoL (*P* = 0.60), than combination probiotics. The individual IBS symptoms exhibited no improvement as a result of the administration of either single or combination probiotics. The advantages of multi- or mono-species probiotics for IBS patients are still inconclusive. Yoon et al. proposed that multi-species probiotics may produce a variety of beneficial effects on IBS symptoms because each species exerts a distinct action on the gastrointestinal tract, and two or more probiotic species in combination may exert a synergistic effect [43]. However, studies have also demonstrated that competition between ingested species or strains may occur, leading to negative effects [46]. The number of RCTs investigating the effects of single probiotics is small, and additional evidence is needed to confirm the superiority of multi-species probiotics.

In the probiotic dose subgroup, both low and high doses were associated with an improvement in the overall symptom response and QoL, but not in individual IBS symptoms. Vicente et al. compared the effects of 2 doses (the high dose, 1-3 × 10^10^ CFU/D, and the low dose, 3-6 × 10^9^ CFU/D) of a new combination of probiotics on the IBS response rate and QoL and reported similar results [45]. This lack of an observed dose effect may be due to the small distinction between the high and low doses [45]. More evidence is needed to confirm the differences between high and low doses.

We also revealed that a short treatment duration (<8 w) may be more effective than a long duration (≥8 w) in improving overall symptom response and QoL. IBS is a chronic and relapsing condition, and the type and severity of symptoms may vary in the same patient over time; longer term or even continuous supplementation of probiotics may be required to detect significant alterations in symptoms [20]. A short treatment duration appeared to be more effective according to the currently available results. Roberts et al. also demonstrated greater improvement with a short duration of treatment, but the large number of dropouts in the long-duration group may have influenced these results [42].

One of the strengths of the current study is that this is the first systematic review and meta-analysis to use Rome III as the IBS diagnostic criteria. The Rome III criteria can be easily applied in clinical practice and research settings and may more accurately reflect disease burden and epidemiological features of the disorder than the Rome II criteria [25]. Another advantage of the current study is the analysis of subgroups of probiotic type, dose, and treatment duration. We also attempted to contact the authors of potential studies to gain access to all of the available data, and more than 1000 IBS patients were included in the current meta-analysis.

There are several limitations in our study. Due to the lack of available studies, neither the effects of individual probiotic species nor the effects of IBS subtypes on IBS patients were analysed. Significant heterogeneity existed due to the various outcome assessment criteria and probiotic types, doses, and treatment durations used in different studies. An appreciable placebo effect was detected in some studies, which may have minimized the effects of probiotics [54].

## Conclusions

In conclusion, our results demonstrate that probiotic supplementation is an effective therapy in IBS patients. Single probiotics at a low dose and with a short treatment duration appear to be more effective in improving overall symptom response and QoL. Future studies of the effects of probiotics in IBS should focus on probiotic type, strain, dose, and treatment duration.

## Abbreviations

CI, confidence interval; GIQLI, gastrointestinal quality of life index; GSRS, gastrointestinal symptom rating scale; HRQOL, health-related quality of life; IBS, irritable bowel syndrome; IBS-SSS, irritable bowel syndrome-symptom severity score; ITT, intention to treat; MMS, mean symptom composite score; QoL, quality of life; RCT, randomized controlled trial; RR, relative risk; SBDQ, standardized bowel disease questionnaire; SMD, standardized mean difference; VAS, visual analogue scale

## Additional files

Additional file 1: Risk of bias. (TIF 107 kb) Additional file 2: Risk of bias summary. (TIF 306 kb) Additional file 3: Funnel plot for publication bias for efficacy of probiotics on the overall symptom response. (TIF 3.38 kb) Additional file 4: Forest plot of effect on abdominal pain of IBS patients to probiotics: subgroup of probiotics type. (TIF 4.39 kb) Additional file 5: Funnel plot for publication bias for efficacy of probiotics on the abdominal pain. (TIF 3.38 kb) Additional file 6: Forest plot of effect on bloating of IBS patients to probiotics: subgroup of probiotics type. (TIF 3.18 kb) Additional file 7: Funnel plot for publication bias for efficacy of probiotics on bloating. (TIF 290 kb) Additional file 8: Funnel plot for publication bias for efficacy of probiotics on QoL. (TIF 3.38 kb)
