# Supplementary figures and images for: Effects of probiotic type, dose and treatment duration on irritable bowel syndrome diagnosed by Rome III criteria: a meta-analysis
Source: BMC Gastroenterol. 2016 Jun 13;16:62. doi: 10.1186/s12876-016-0470-z (PMC4907258; doi:10.1186/s12876-016-0470-z)

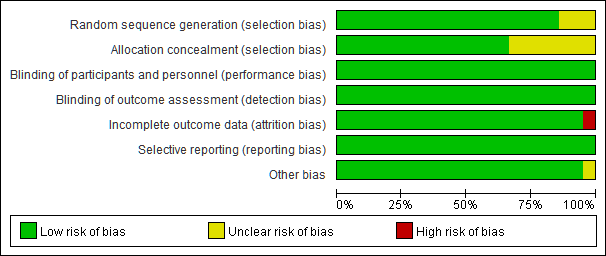

Supplement: Additional file 1: — Risk of bias. (TIF 107 kb) [file 12876_2016_470_MOESM1_ESM.tif]

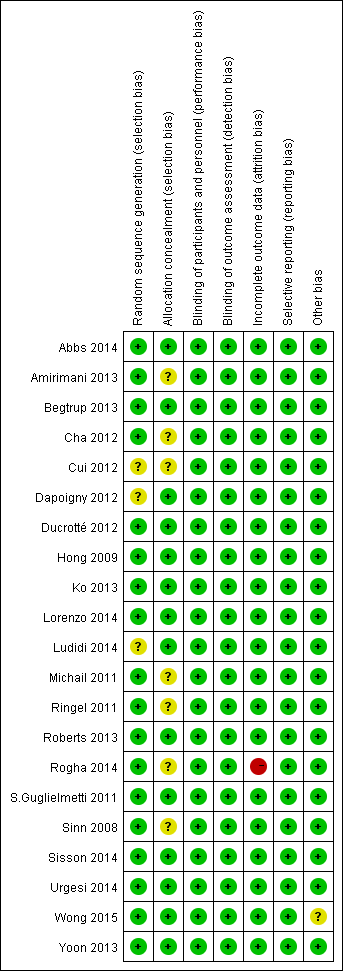

Supplement: Additional file 2: — Risk of bias summary. (TIF 306 kb) [file 12876_2016_470_MOESM2_ESM.tif]

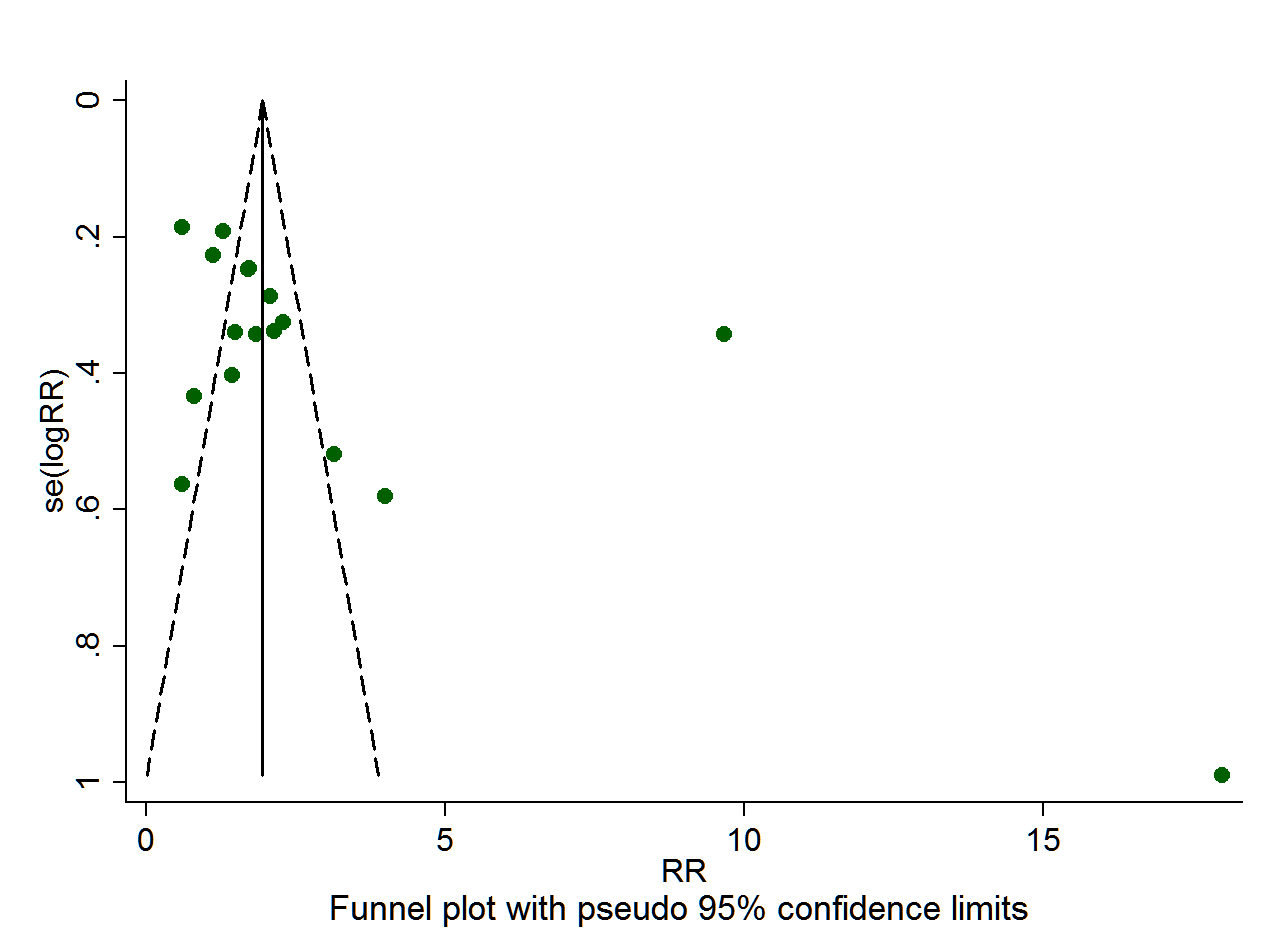

Supplement: Additional file 3: — Funnel plot for publication bias for efficacy of probiotics on the overall symptom response. (TIF 3.38 kb) [file 12876_2016_470_MOESM3_ESM.tif]

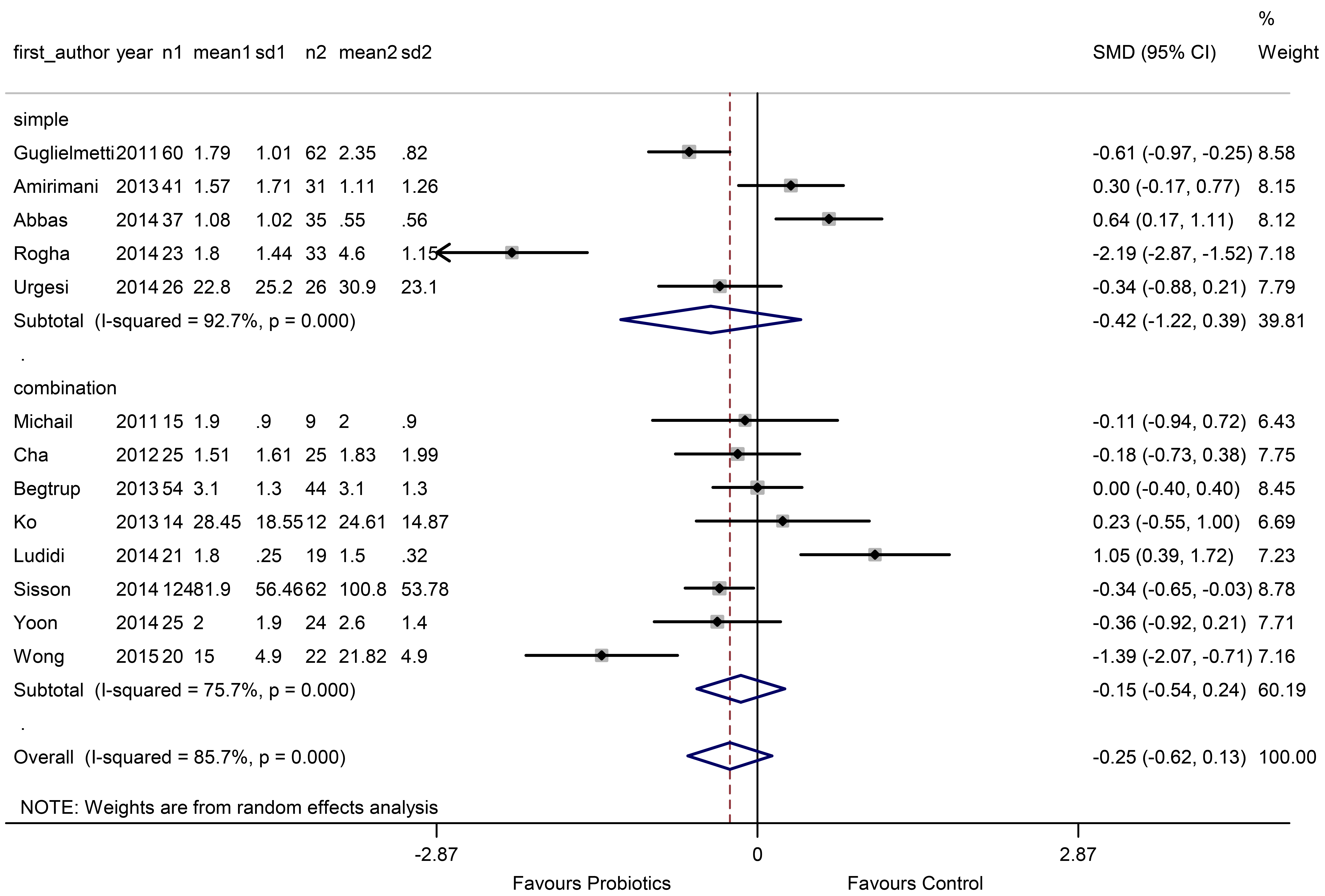

Supplement: Additional file 4: — Forest plot of effect on abdominal pain of IBS patients to probiotics: subgroup of probiotics type. (TIF 4.39 kb) [file 12876_2016_470_MOESM4_ESM.tif]

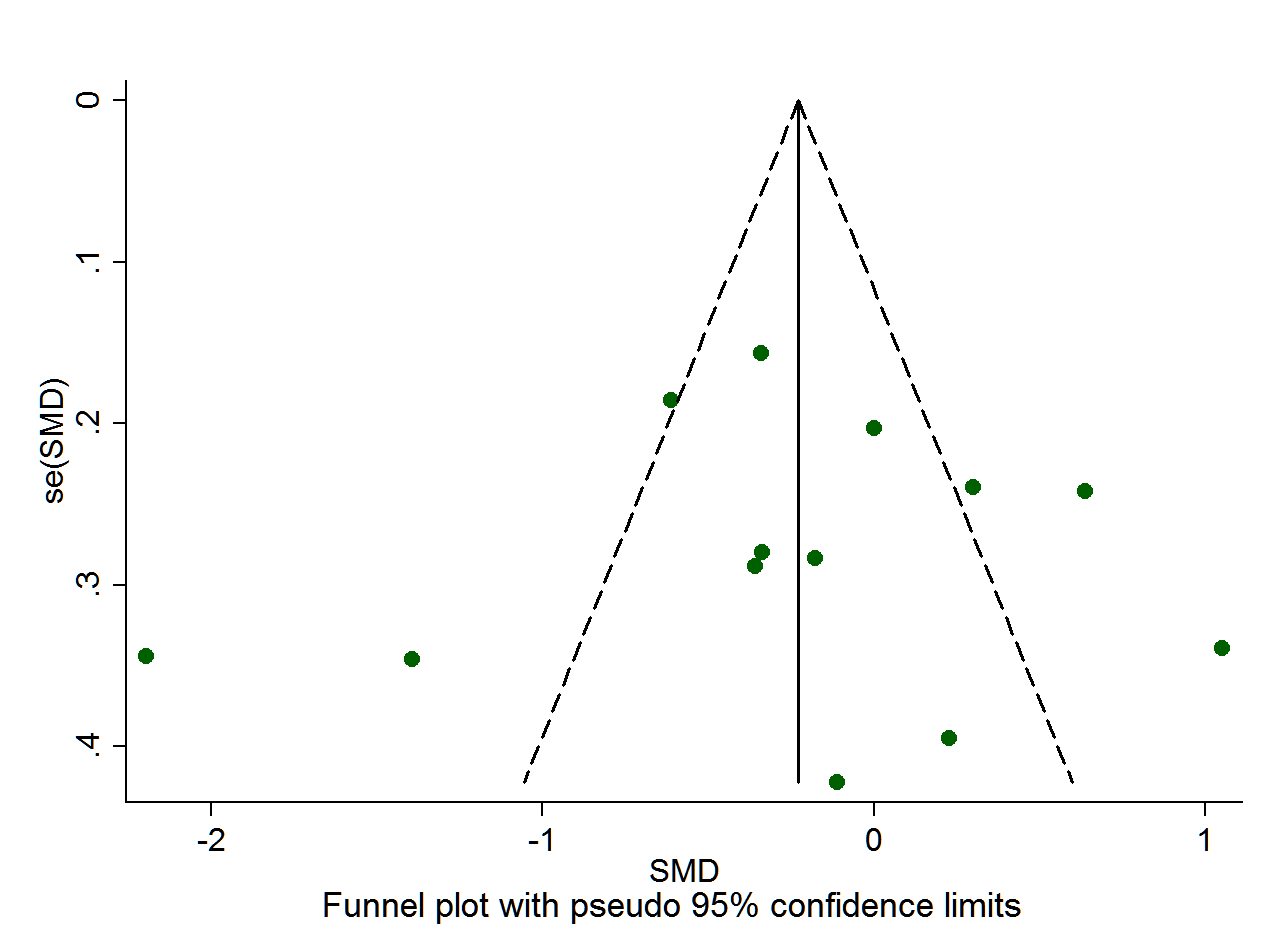

Supplement: Additional file 5: — Funnel plot for publication bias for efficacy of probiotics on the abdominal pain. (TIF 3.38 kb) [file 12876_2016_470_MOESM5_ESM.tif]

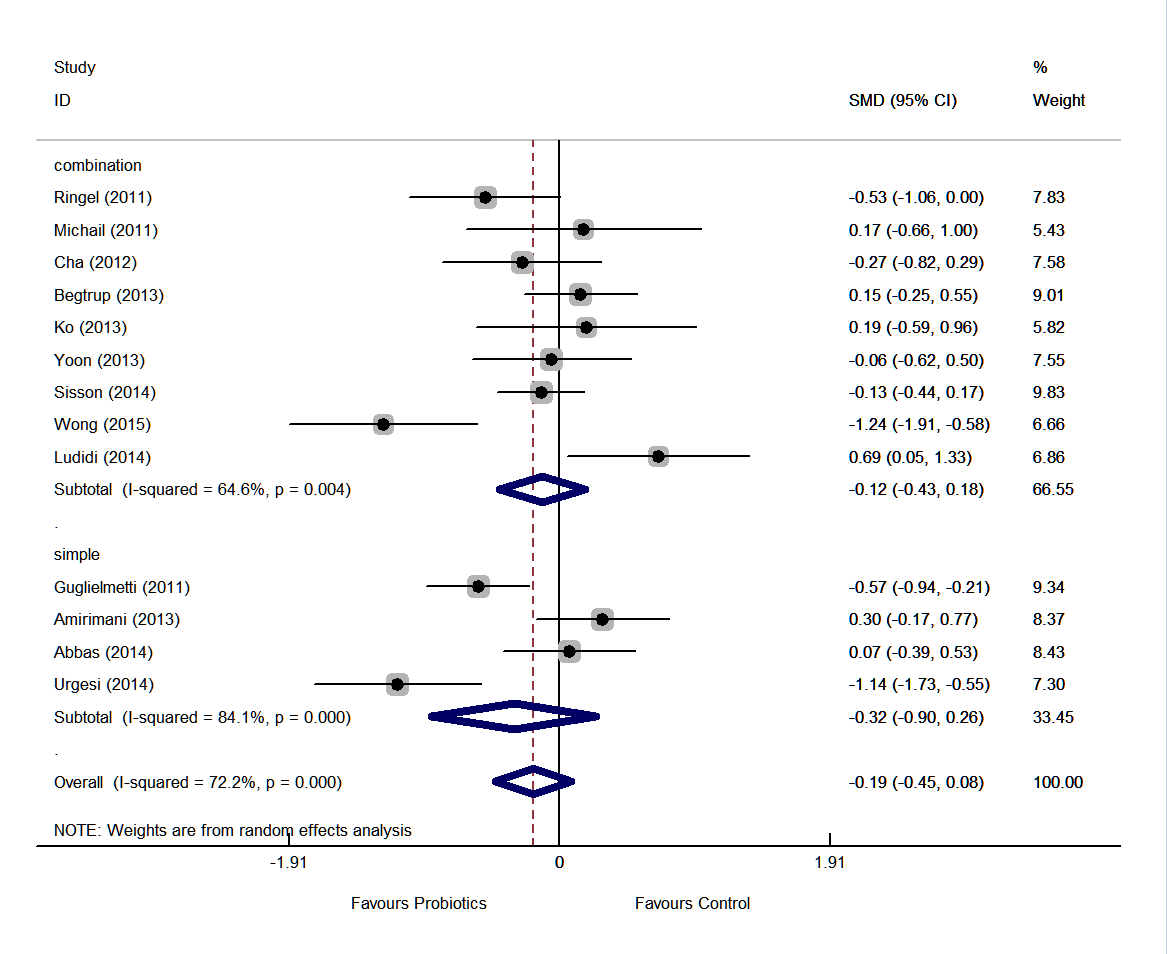

Supplement: Additional file 6: — Forest plot of effect on bloating of IBS patients to probiotics: subgroup of probiotics type. (TIF 3.18 kb) [file 12876_2016_470_MOESM6_ESM.tif]

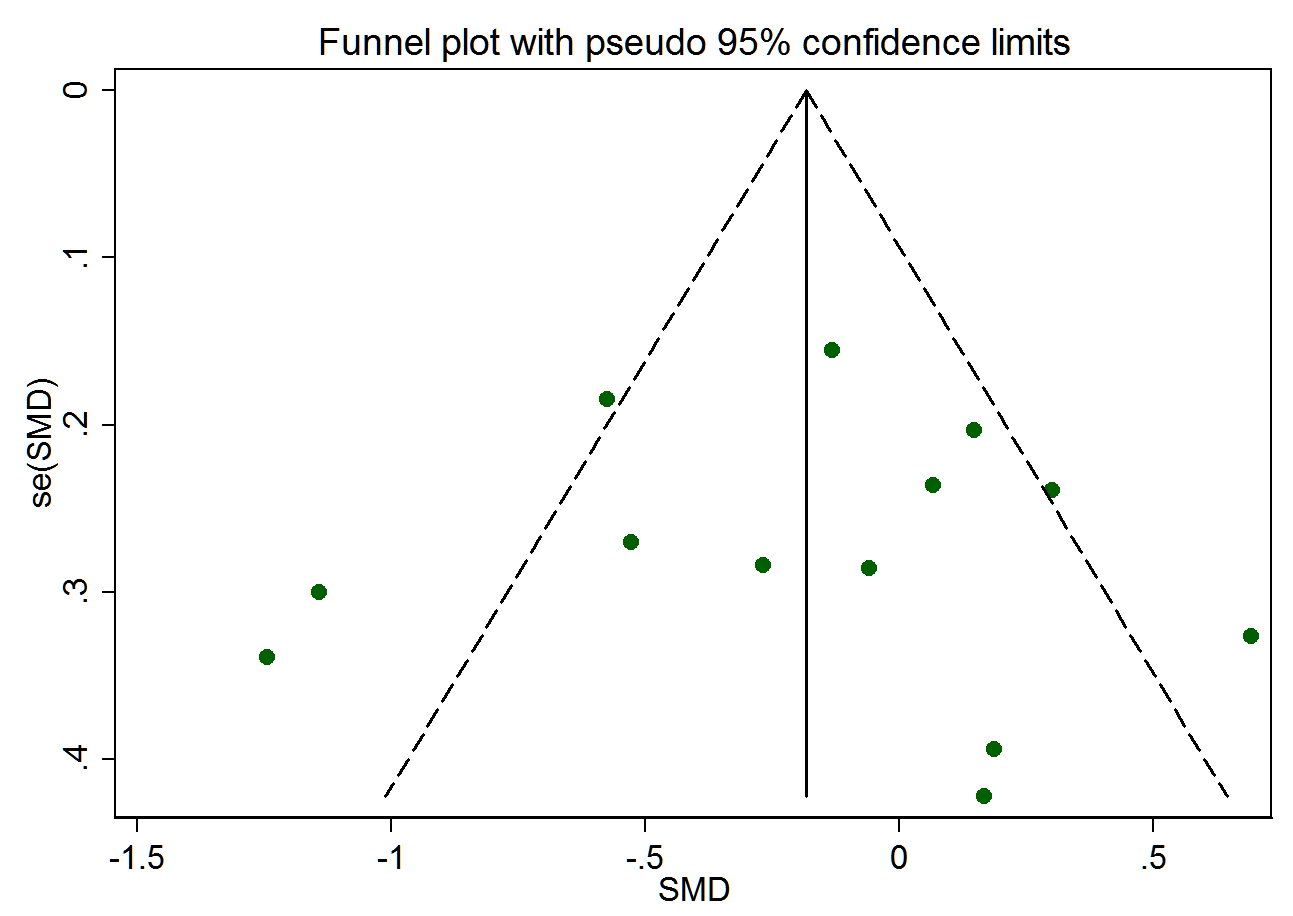

Supplement: Additional file 7: — Funnel plot for publication bias for efficacy of probiotics on bloating. (TIF 290 kb) [file 12876_2016_470_MOESM7_ESM.tif]

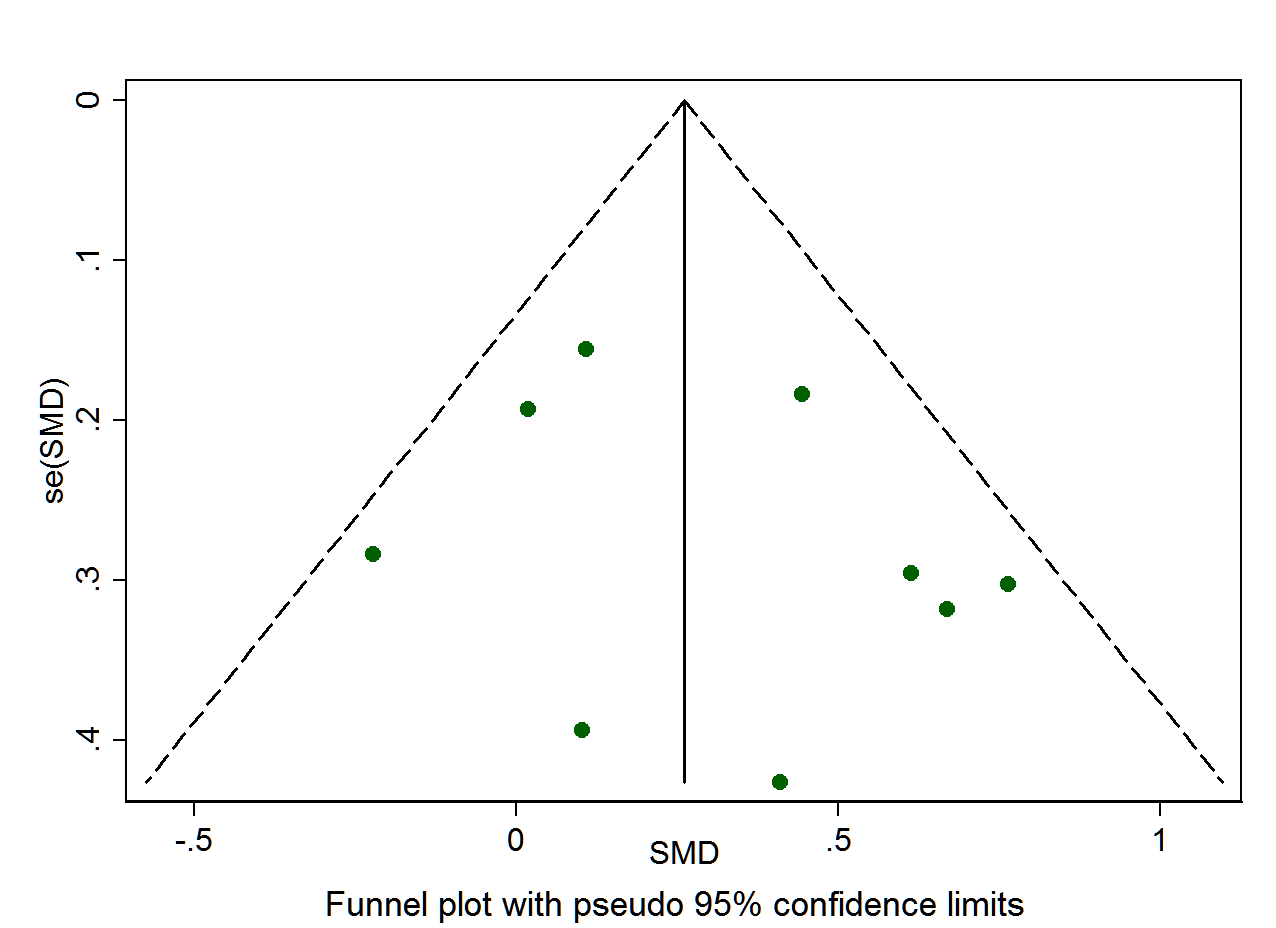

Supplement: Additional file 8: — Funnel plot for publication bias for efficacy of probiotics on QoL. (TIF 3.38 kb) [file 12876_2016_470_MOESM8_ESM.tif]
